# Population-Level Impacts of Alcohol Use on Mental and Physical Health Outcomes

**DOI:** 10.3390/healthcare12161592

**Published:** 2024-08-09

**Authors:** Janet L. Fanslow, Ladan Hashemi, Pauline J. Gulliver, Tracey K. D. McIntosh, David A. L. Newcombe

**Affiliations:** 1 Social and Community Health, School of Population Health, Faculty of Medical and Health Sciences, University of Auckland, Auckland 1023, New Zealand; 2 Māori Studies and Pacific Studies, Faculty of Arts, University of Auckland, Auckland 1010, New Zealand

**Keywords:** alcohol use, drinking patterns, health outcomes, positive mental health, New Zealand

## Abstract

This study explores patterns of alcohol drinking within a representative New Zealand sample (2887 participants (1464 female, 1423 male)). Alcohol use and drinking patterns across the population are described. Multivariable logistic regressions document associations between alcohol use and drinking patterns and the likelihood of experiencing different health outcomes. Alcohol use, early drinking initiation, frequent drinking, and heavy episodic drinking (HED) are prevalent in New Zealand and vary in relation to gender, age, and socioeconomic characteristics. Those who reported alcohol-related problems were more likely to report poor mental health (AOR: 2.21; 95% CI: 1.42–3.46) and disability (AOR: 1.79, 95% CI: 1.06–3.00), and less likely to experience positive mental health (AOR: 0.28, 95% CI: 0.18–0.42). Those who reported HED were also less likely to experience good general health (AOR: 0.61, 95% CI: 0.47–0.81) and positive mental health (AOR: 0.67, 95% CI: 0.53–0.84). Younger age cohorts were more likely to engage in early drinking, and those who initiated regular drinking before age 18 were more likely to report HED and alcohol-related problems. Findings indicate that problem drinking and HED are not only associated with poor physical health, but also reduce the likelihood of individuals experiencing positive mental health. This provides information to enable public health practitioners to target alcohol prevention strategies at the entire population of drinkers.

## 1. Introduction

Alcohol is one of the primary risk factors for the global health burden [1,2], accounting for 4.7% of deaths and causing 115.9 million disability-adjusted life years (DALYs) in 2019 (4.6% of all DALYs) [2]. While alcohol consumption is determined by a host of factors (biological, psychological, and social), there is also evidence that populations tend to behave as collectives, moving up and down the consumption scale [3]. Regular monitoring of alcohol use in the population and within population sub-groups has been deemed essential to observe trends over time and for developing appropriate policy to minimise alcohol-related harm [4,5].

Patterns of drinking (such as frequent drinking, heavy episodic drinking (HED), and early drinking initiation (EDI)) are associated with an increased risk of adverse health outcomes [5]. HED has been linked to serious consequences for health (e.g., cardiovascular and liver disease) [6] and safety (e.g., impaired driving and injuries (both unintentional and intentional)) [7]. EDI is also a predictor of impaired health, as it increases the risk for alcohol dependence and abuse at later ages [8]. While there is convincing evidence that the developing brain is vulnerable to the harmful effects of alcohol [9], many of the long-term consequences of EDI are not completely understood [10].

Previous studies have documented the association between alcohol drinking and later mental health but have defined mental health as “fewer problems” or “the absence of mental illness”. However, mental health can also be measured as a ‘positive state’, defined as optimal psychological functioning and a general feeling of well-being [11,12]. Using this conceptualisation and measuring mental health as a positive state provides opportunities to move beyond a deficit model, as positive mental health is associated with a range of health benefits [11,12]. Exploring indicators of both positive mental health and indicators of ill health may enable us to develop a more holistic understanding of the impacts of drinking behaviours.

Findings from two New Zealand surveys provide national evidence on alcohol and patterns of drinking [13,14,15]. These nationally representative surveys indicate that New Zealand has a very high rate of alcohol consumption [13], but they do not fully capture the breadth of health consequences associated with alcohol use. Population trends in EDI have also not been explored.

In this paper, we use data from a large population-based New Zealand survey to examine (a) prevalence of alcohol use, HED, and EDI, (b) the association of drinking patterns and markers of health outcomes (both positive and negative), (c) associations between EDI and alcohol drinking patterns, and (d) changes in age of drinking initiation.

## 2. Materials and Methods

### 2.1. Data Source

Data were obtained from He Koiora Matapopore, a population-based, cross-sectional retrospective survey conducted in three regions in the northern half of the North Island (Waikato, Northland, and Auckland) in New Zealand between March 2017 and March 2019. These regions account for approximately 40% of the New Zealand population and include rural and urban locations and a diverse mix of Māori, Pasifika, Asian, and European New Zealanders. The survey collected data from individuals aged 16 and over who were able to speak English and answer questions on a wide range of life experiences, including health risk behaviours (e.g., alcohol use), and physical and mental health outcomes. Full details of the study methods are published elsewhere [16] but are summarised here.

### 2.2. Data Collection and Sampling Method

A simple random sampling approach was used to obtain a survey sample representative of the population of the regions represented. Primary sampling units (PSU) based on meshblock boundaries were used to conduct random sampling. These units are the smallest geographical units used by Statistics New Zealand [17]. Within each meshblock, a random starting point was identified, and every second and sixth house within the meshblock was selected. Non-residential and short-term residential properties, rest homes, and retirement villages were excluded. Specific meshblocks were allocated to each gender for safety reasons. Only one randomly selected person per household could participate in the study.

Comprehensive training of all interviewers was conducted to ensure valid data collection, and the safety of interviewers and respondents. Face-to-face interviews were conducted privately with no one aged 2 years or over present. All respondents provided written consent prior to the interview. Ethics approval was received from The University of Auckland Human Participants Ethics Committee (reference number 2015/018244).

### 2.3. Study Sample

**Figure 1:**
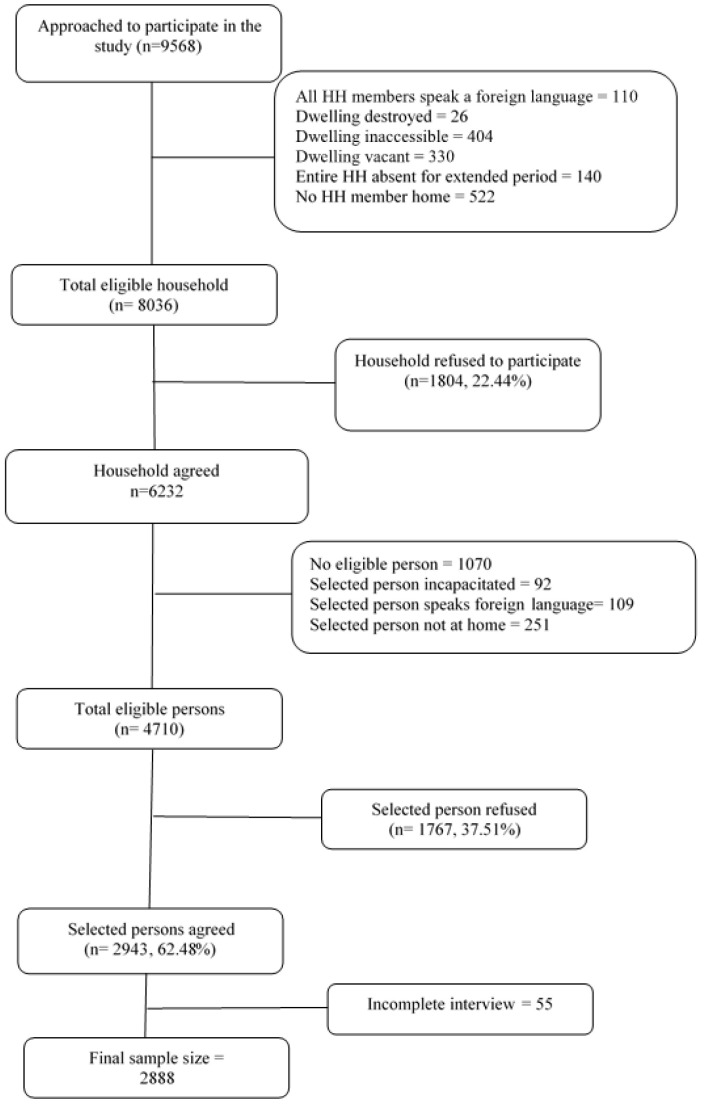
He Koiora Matapopore participant recruitment and inclusion flowchart. Reprinted from Child Abuse & Neglect, 117/105067 [18].

Of 9568 approached households, 1532 were ineligible to participate. Of these, the largest number who were ineligible to participate occurred as a result of not being present at the house after multiple visits to recruit participation. There were also a number of dwellings that interviewers could not gain access to. In general, these were apartments within an apartment building with restricted access (see Figure 1).

Of 8036 eligible households, 1804 (22.4%) refused to participate. Of 6232 households who agreed to participate, 1271 participants were ineligible (mainly due to not speaking English or being incapacitated). A further 251 were not at home after multiple interviewer visits. Of the remaining 4710 eligible participants, 1767 (37.5%) refused to participate. After excluding incomplete interviews (n = 55), 2888 participants remained in the study (1464 female and 1423 male, and 1 other (excluded from analyses)), yielding an overall response rate of 63.7% (Figure 1). Sociodemographic characteristics of the study sample are presented in Table 1.

Representativeness: The ethnicity, marital status, and deprivation level distribution of the sample were closely comparable to the general population of New Zealand; however, the sample was under-represented for younger people (ages 16–29) and slightly over-represented for those over 60 years of age. See [16] for details.

### 2.4. Measures

#### 2.4.1. Sociodemographic Characteristics

Prevalence rates of reported alcohol use among sub-groups of the population were explored based on sociodemographic characteristics and to identify potential confounders to be included in multivariable analyses. Variables included age (16–24 years, 25–34 years, 35–44 years, 45–54 years, 55–64 years, and 65 and over years), ethnicity (European, Māori, Pasifika, Asian, and MELAA (Middle East, Latin American, African)), access to an independent source of income, personal income level (less than median income (<NZD 50K), more than median income (≥NZD 50K)) [19], area-level deprivation taken from the NZ Index of Multiple Deprivation (IMD) [20], and food security status (see Supplementary Table S1).

#### 2.4.2. Alcohol Use, Drinking Patterns, and Alcohol-Related Problems

Four measures were used to define alcohol-related problems: early drinking initiation (EDI), high-frequency drinking, heavy episodic drinking (HED), and problems related to alcohol use and substance abuse disorder. Questions regarding the frequency and quantity of drinking were asked. The frequency of drinking was established by asking how often each respondent drank alcohol. Frequent drinking was defined as drinking every day or nearly every day. Quantity of drinking was then assessed by inquiring about the typical number of alcoholic drinks consumed on the days when the individual drank during the past four weeks. Those who had never drunk in their life (n = 438) and those who indicated they did not currently drink (drank in the past, not now; n = 184) were not asked the question about the quantity of consumption and were excluded from analyses of the quantity variable (drink per occasion).

Following the New Zealand Health Promotion Agency guidelines [13], HED was defined as four or more (for female respondents) or five or more standard drinks (10 g of pure alcohol; for male respondents) on the days that the respondent drank in the past four weeks. For HED and frequent drinking, those not currently drinking were assigned a zero and were included in the analyses. Enquiries were also made about the age at which regular drinking was initiated (EDI). Alcohol-related problems were defined as self-reported problems “related to your drinking” (in the past 12 months). The types of problems included money, health, conflict with family or friends, or problems with authorities. Supplementary Table S1 provides definitions for each alcohol measure.

#### 2.4.3. Outcome of Interest: Health Status

Adverse health outcomes were measured through self-reports of clinically diagnosed chronic physical health conditions, poor mental health, and disability. Positive health outcomes were measured through self-perceived ratings of positive mental health using Keyes’ Mental Health Continuum Short Form (MHC-SF) [11] and good general health (Supplementary Table S1).

### 2.5. Analytic Procedures

All analyses were conducted using Stata 15.1 [21] survey commands to allow for stratification by sample location (three regions), clustering by primary sampling units (PSU), and weighting of data to account for the number of eligible participants in each household.

Problems with missingness were minor, around 1% for alcohol variables. Descriptive statistics (percentages with 95% confidence intervals (CIs)) were used to describe the prevalence of each sociodemographic characteristic for the whole sample and by gender (Table 1). Prevalence rates and 95%CIs, for the whole sample and stratified by gender and other sociodemographic characteristics, were also calculated for each alcohol-related measure (Table 2 and Table 3).

Chi-square tests were used to examine whether alcohol-related variables differed by gender and other sociodemographic characteristics. Means and standard deviations were reported for age of onset of regular drinking. An independent *t*-test was used to determine whether there was a statistically significant difference in mean age at regular drinking onset between male and female participants.

Logistic regressions were run to calculate the odds of experiencing both adverse and positive physical and mental health outcomes by binary alcohol-related measures, adjusted for established sociodemographic predictors of health outcomes that had significant associations in bivariate analysis at *p* < 0.05 (i.e., gender, age, ethnicity, area deprivation level, food security status, having an independent source of income, and personal income level). Results were reported as adjusted odds ratios (AORs) with 95% CIs. Unadjusted odds ratios (ORs) with 95% CIs were also reported.

Finally, to determine whether the association between alcohol-related measures and health outcomes found in the previous step were consistent across genders, multivariate logistic regression models with interaction terms (between each alcohol-related measure and gender) were tested. Potential confounders (e.g., age, ethnicity, area deprivation level, food security status, having an independent source of income, and personal income level) were also included in these analyses.

## 3. Results

### 3.1. Prevalence of Alcohol Drinking Measures for the Whole Sample

The average age reported at first regular drinking for the whole sample was 20.4 years old (SD = 7.94). Using the binary variable for EDI (under the legal age of 18 years for purchasing alcohol), it was reported by 44.6% of the sample. Age cohorts within the sample showed a progressively earlier mean age of initiating regular drinking, with the youngest group reporting the earliest age of regular drinking initiation (16.8 years), compared with the oldest cohort (those aged 65 years and over had an average age of 23.4 years for regular drinking initiation; Table 4).

Of the whole sample, 24.3% reported that they were not current drinkers, consisting of 17% who reported that they had never drunk alcohol and 6.4% who reported that they had drunk in the past but not now. Frequent drinking, defined as every day or nearly every day, was reported by 18.9% of the sample. HED was reported by 19.5% of the sample.

### 3.2. Gender Differences in Alcohol Drinking Measures

Men initiated regular drinking on average two years earlier than women (19.5 years for men and 21.5 years for women), and a significantly higher proportion of men reported EDI and frequent drinking compared with women (Table 2).

### 3.3. Alcohol Drinking Measures by Sociodemographic Characteristics

EDI was less common among those age 65 years and older compared with younger groups. However, this group were more likely to report frequent drinking (32%) but less likely to report HED (10.3% for ≥65 versus 31% for 16–24 years old).

High proportions of both Māori and European participants reported EDI (55.7% and 51.0%, respectively). HED was most frequently reported by Pasifika (33.9%) and Māori (32.5%) participants. Over half of those who identified as European (51%) reported EDI, and Europeans also had the highest proportion of frequent drinking (26.3%).

Those who had higher socioeconomic status (i.e., earned an income more than NZD 50K, lived in areas with low deprivation, and those classified as food secure) reported higher proportions of EDI, frequent drinking, and substance abuse disorder. Those classified as food insecure reported higher proportions of HED and alcohol-related problems, compared with those classified as food secure.

### 3.4. Health Consequences of Alcohol Behaviours

After adjusting for sociodemographic factors, HED was significantly associated with a decreased likelihood of experiencing both good general health and positive mental health. There were no significant associations between EDI, frequent drinking, and adverse or positive self-reported health outcomes. When interaction terms were included in the multivariable logistic regressions, no significant interaction effects were found between alcohol use measures and gender for any health outcome (Table 5). The only exception was for poor mental health, where significant interactions were found between frequent drinking (*p* = 0.006), HED (*p* = 0.01), and gender, with women more likely to report poor mental health than men.

While EDI was not associated with health outcomes, it was associated with a higher likelihood of reporting HED and alcohol-related problems (Table 6).

## 4. Discussion

This study reports on the prevalence of alcohol use and drinking patterns using data from a large population-based New Zealand study. Distribution of alcohol use and drinking patterns across gender, age, and key socioeconomic sub-groups were also explored. Those who reported HED were more likely to report poor mental health and disability, and less likely to experience positive mental health. Those who reported HED were also less likely to experience good general health and positive mental health.

### 4.1. Prevalence of Alcohol Use and Alcohol Drinking Patterns

In this population-based sample, three out of four (75%) New Zealand adults aged 16 and over reported current consumption of alcohol, and 45% reported drinking once or more per week. This is comparable to the 78.5% of those aged 15 years or over who reported having consumed alcohol in the past 12 months, reported by the New Zealand Health Survey (NZHS) [15], and below the 83% reported by Alcohol Use in New Zealand survey 2019/2020 (AUINZ) [13]. These estimates might not be directly comparable due to differences in question wording. For example, both NZHS and AUINZ asked about having an alcoholic drink in the past 12 months, while the question used in the current survey did not have a restricted timeframe.

The prevalence of current alcohol consumption found in the present study is higher than the rate of consumption reported in the US (69.5%) [22], but lower than the rates reported in the Australia (79%) [23] and UK (82%) studies [24], although these figures might not be directly comparable due to differences in variable definitions.

In this survey, almost one in five (18.9%) reported frequent drinking (every day or nearly every day), and the same proportion reported HED. This is substantially lower than the 33% that was reported in the 2019/2020 AUINZ survey, which reports on the prevalence of exceeding the single-occasion limit suggested to reduce the health risks of consuming alcohol (four standard drinks for women and five standard drinks for men) [13]. This is likely to be the result of differences in the questions about quantity, including provision of the definition of a standard drink in the question in the AUINZ survey, as this specificity of quantity was not included in the current investigation.

### 4.2. Gender Differences

Men were more likely than women to report that they had drunk alcohol within the last week and were more likely to report EDI and frequent drinking. A similar finding was reported by the AUINZ survey, where men were 10% more likely to be a past-week drinker than women (60% for men vs. 50% for women) [13]. This finding was also consistent with results from the 2018/2019 NZHS, which found that men were twice as likely as women to be hazardous drinkers [14].

A gendered pattern in alcohol consumption has been reported in other countries (e.g., Australia, the US, and South Korea) [25,26]. Explanations for gender differences in alcohol consumption include biological differences in alcohol metabolism between men and women and higher exposure opportunities among men due to psychological, family, and social factors [27]. Evidence of the gender differences found in this study echoes the need for policies and services related to women’s and men’s alcohol consumption to have a gendered focus.

### 4.3. Age Differences

The average age of initiation of regular drinking dropped progressively between age cohorts—there was almost a seven-year difference in the mean age of drinking initiation between the oldest and the youngest age cohorts. Frequent drinking was more prevalent among the oldest group (65+ years old), while HED was more prevalent among the youngest group (16–24). The high prevalence of HED among the youngest group was consistent with the findings of the NZHS 2018/2019, which found that younger groups (those aged 18–34 years old) had the highest prevalence of hazardous drinking [14]. These findings warrant attention, as younger people may engage in more unsafe behaviour while drunk (e.g., unsafe driving [28]).

Our findings were also in line with the World Health Organization’s Global Status Report on Alcohol and Health, indicating that while alcohol consumption generally declines with age, older drinkers typically consume alcohol more frequently than other age groups [5]. White and colleagues have identified this as a potential public health issue, with the growing aging population and changes in physiology resulting in higher blood alcohol concentration and more impairments in behaviour and cognition with age [29].

Monitoring of alcohol consumption and policy responses might be required to address age-specific vulnerabilities, including the potential for alcohol and medication interactions in older people.

### 4.4. Socioeconomic Differences

Frequent drinking was more prevalent among those with higher socioeconomic status (i.e., those having access to an independent source of income, or who earned an income more than NZD 50K, or who lived in areas with low deprivation, and those classified as food secure). HED was more prevalent among those classified as food insecure. These findings are consistent with those from the 2018/2019 NZHS, which found that, despite fewer people in the most deprived areas having drunk alcohol in the past year, adults in the most deprived areas were 1.3 times more likely to be hazardous drinkers than adults in the least deprived areas [14].

These findings are consistent with the ‘alcohol harm paradox’ [30], a concept used to describe the higher prevalence of experiencing alcohol-related harm reported by those from lower socioeconomic status groups, while individuals in higher socioeconomic status groups were more likely to report exceeding the recommended drinking limits. These differences in drinking patterns highlight that policies that seek to address alcohol-related health inequalities need to consider that the extreme drinking levels in some sub-groups may be associated with multiple markers of deprivation. This will require a more nuanced understanding of drinking practices in these vulnerable sub-populations [31,32].

### 4.5. Ethnicity

Frequent drinking was more prevalent among those who identified as European, while HED was more prevalent among those who identified as Māori or Pasifika. Those who identified as Asian had the lowest prevalence rate for all measures of alcohol use, followed by the MELAA group. These findings are in line with those from the 2018/2019 NZHS, which found that Māori and Pasifika people are more likely to be classified as hazardous drinkers than other ethnic groups [14]. These differences might be partially explained by socioeconomic differences among ethnic groups, where those who identify as European are more likely to have a higher socioeconomic status than Māori or Pacific people. There is also a link between hazardous drinking and experiences of racial discrimination [33,34]. These findings lend support to the calls for the availability of culturally appropriate treatment services [35].

### 4.6. Association between EDI and Later Alcohol Drinking Patterns

EDI was common, with 45% of the sample reporting that they started regular drinking under the legal age for purchasing alcohol of 18 years. EDI was associated with a higher likelihood of frequent drinking and HED. These findings are consistent with evidence indicating that EDI is associated with the development of alcohol problems [36,37]. For example, a Chilean study found that youth who start drinking alcohol later (at age 16 or over) were less likely to engage in heavy episodic drinking (HED) [38]. Collectively, this evidence supports an argument for delaying alcohol initiation to reduce the risk of alcohol-related problems in later life. Increasing the price of alcohol products may delay alcohol initiation among young people.

### 4.7. Health Consequences of Alcohol Behaviours

HED decreased the likelihood of experiencing good general health and positive mental health. This finding is consistent with those from other studies among a sample of US adults [25] and a Swedish population attending primary healthcare [39], suggesting that binge or “risky” drinking is more detrimental to health than frequent drinking. Frequent drinking increased the likelihood of experiencing poor mental health, but only for women. These findings underscore the need for interventions and alcohol controls as an important policy strategy for reducing alcohol use and to mitigate its harmful consequences among New Zealanders.

Of note, we did not ask that respondents report their alcohol intake according to ‘standard drink size’ (measured as 10 g of alcohol in New Zealand). Instead, alcohol consumption was assessed according to how many drinks were consumed per week or on an average occasion (see Supplementary Table S1). While this may introduce some variability in responses depending on drink size, previous investigations have shown that drinkers struggle to understand standard drink sizes [40].

### 4.8. Limitations

The cross-sectional nature of this study means a temporal relationship between alcohol use and the onset of health outcomes could not be inferred. However, the consistency of findings assessing different measures of alcohol drinking patterns and health outcomes and the articulation of causal pathways by which alcohol use can influence health that have been identified through other studies may mitigate this concern [41,42,43]. Findings from World Mental Health Studies have highlighted the strong association between alcohol use disorders and ‘common mental disorders’. Of note from cross-national studies was that other mental disorders most often preceded the onset of alcohol use disorders [44]. These findings highlight the need to treat alcohol use and other mental health conditions in parallel.

The present study found non-significant associations between alcohol drinking patterns and chronic physical health conditions. Selection bias may have contributed to this finding, as, for example, those classified as a HED drinker or being a frequent drinker may have been less likely to be included in our sample because they were too ill to participate, they needed institutional care, or they died at younger ages [45]. The under-sampling of heavy drinkers has been noted in observational studies across the globe. At the population level, recommendations are made to uplift survey estimates in order to accommodate for the under-coverage of heavy drinkers [46]. Alternatively, recruitment methods that target primary care, or include community outreach strategies, have been found to improve the recruitment of heavy drinkers [47].

Use of self-report measures for exposure and outcome variables also have limitations, as they were not independently verified. Alcohol consumption estimates were measured using self-report data, which is likely to underestimate the consumption volume [48]. Social desirability may also have affected responses, as participants may be particularly disinclined to report problematic alcohol drinking to a live interviewer. As highlighted above, where there is a desire to specifically understand the role of heavy alcohol consumption on health outcomes, we recommend the use of alternate recruitment strategies. We note, however, that the use of primary healthcare as a recruitment method would limit the study population to those who are currently seeking help for their alcohol consumption. Flor and Gakidou have underscored the difficulties associated with obtaining accurate estimates of alcohol consumption [49]. To address this, they recommend the use of risk factor surveillance systems to measure unrecorded alcohol use. We recommend a cautious approach in using such systems as an alternate measure of problem alcohol consumption, noting how the use of tools such as risk analysis and predictive risk modelling have the potential to embed inequities [50].

The definitions used in the present study about alcohol consumption and patterns of use were general, and questions did not always operationalise the specific aspect of the measure as per industry standard, e.g., we did not specify standard drink size when asking about the quantity of alcohol consumed.

Further, this study was limited to the geographical location in which it was conducted (the northern-most region of New Zealand) and to those who chose to participate. We anticipate that those who took part in the investigation were, in general, healthier. Further, limiting survey participants to those who could hold a conversation in English is likely to result in an under-representation of recent migrants to the country. As such, the findings presented within this investigation cannot be considered representative of New Zealand as a whole.

### 4.9. Strengths

This investigation added to the literature concerning the association between alcohol consumption and health outcomes by investigating the frequency, heavy episodic nature, and early initiation on health outcomes. This study extended previous work by assessing a full range of alcohol drinking patterns and documented the association between drinking patterns and health outcomes. Additionally, this study also extended previous work by looking at a comprehensive range of health outcomes, including poor physical and mental health, disability, and indicators of positive health. This is unique internationally, as most previous studies have explored the effects of alcohol use on limited types of health outcomes.

### 4.10. Implications

These findings reinforced previous calls for the need for initiatives that can mitigate the deleterious consequences of alcohol consumption at the population level. This could be more cost-effective than strategies that seek to alter the behaviours of individuals, particularly after they have developed problematic patterns of alcohol use. The associations found between alcohol drinking patterns and health outcomes are relevant to the health of the nation. Development and implementation of effective alcohol harm prevention programs could limit the development of later adverse outcomes, thus reducing individual pain and suffering and preventing socioeconomic and healthcare-related costs to society.

Policies that restrict the availability and promotion of alcohol have been shown to be effective in reducing both alcohol consumption and alcohol harm [51,52,53,54], with benefits of strategies such as reducing alcohol outlet densities reducing alcohol-related harms regardless of SES [55]. However, commentators highlight the lag in policies targeting the harms associated with alcohol use when compared with the significant effort placed on addressing other ‘dependence-producing’ substances, such as tobacco [49]. They point to a need for a multiplicity of strategies aimed at reducing the harms associated with alcohol, including taxation, availability of treatment options, and policies impacting pricing, advertising, and accessibility. Comprehensive alcohol harm prevention strategies should also include efforts to reach at-risk consumers, including older adults, and ensure widespread availability of accessible and acceptable treatment options, especially for indigenous and minoritised populations [56]. Public health policies that can prevent or delay initiation of drinking are also warranted, given the potentially long-lasting consequences of early alcohol consumption.

## 5. Conclusions

This study reported on the prevalence of alcohol use and alcohol drinking patterns in New Zealand. It provided evidence that HED was associated with increased odds of experiencing poor mental health and decreased odds of experiencing good general health and positive mental health. There is a need for comprehensive public health alcohol harm prevention strategies targeted at the entire population of drinkers, including efforts aimed at subgroups of high-risk drinkers.

## Figures and Tables

**Table 1:** Sample demographic and socioeconomic characteristics: total sample and by gender.

	Total N (%)	Male N (%)	Female N (%)
	2887	1423 50.2 (45.5–54.8)	1464 49.8 (45.2–54.4)
Age groups *			
16–24	228 (12.8)	129 (14.7)	98 (10.8)
25–34	355 (13.5)	165 (13.6)	190 (13.3)
35–44	507 (16.0)	268 (16.5)	239 (15.5)
45–54	548 (19.7)	270 (18.8)	278 (20.7)
55–64	508 (16.3)	252 (16.6)	256 (16.0)
≥65	738 (21.7)	337 (19.8)	401 (23.6)
Ethnicity *			
European	1984 (63.0)	963 (61.8)	1021 (64.2)
Māori	318 (12)	129 (9.3)	188 (14.5)
Pasifika	159 (7.9)	88 (8.1)	71 (7.7)
Asian	378 (15.7)	218 (19.1)	160 (12.2)
MELAA	45 (1.5)	23 (1.6)	22 (1.3)
Having independent source of income *			
No	492 (20.8)	173 (16.6)	319 (24.9)
Yes	2393 (79.2)	1248 (83.4)	1144 (75.0)
Individual income *			
<NZD 50K	1504 (57.0)	573 (45.7)	930 (68.8)
≥NZD 50K	1230 (43.0)	801 (54.3)	429 (31.2)
Deprivation level *			
Low	789 (25.8)	365 (24.4)	424 (27.2)
Moderate	1243 (41.8)	648 (44.3)	595 (39.3)
High	852 (32.4)	410 (31.3)	442 (33.5)
Food security *			
Secure	2352 (81.1)	1183 (83.1)	1169 (79.2)
Insecure	519 (18.9)	230 (16.9)	289 (20.8)

* The n in each category may not add up to the total N, due to missing data.

**Table 2:** Distribution of alcohol use measures in the total sample and by gender.

	Total Mean (SD)	Male Mean (SD)	Female Mean (SD)	*t*-Test (*p*-Value)
**Age at regular drinking onset**	20.43 (7.95)	19.46 (6.43)	21.46 (9.17)	*t*-test (1927.3) = 5.93 (<0.001)
	**Total** **n (W%)**	**Male** **W% (CI)**	**Female** **W% (CI)**	***p*** **-value for Chi-square**
**Early drinking initiation**	1283 (44.6)	48.5 (45.2–51.7)	40.7 (37.7–43.8)	0.0006
**Frequency of drinking**				<0.001
Never	438 (17.0)	14.4 (12.0–17.1)	19.7 (16.5–23.4)	
Less than once a month	426 (16.0)	13.4 (11.5–15.5)	18.7 (16.4–21.3)	
1–3 times a month	410 (15.6)	16.0 (13.8–18.5)	15.1 (13.1–17.4)	
Once or twice a week	798 (26.0)	28.2 (25.6–30.9)	23.7 (21.4–26.3)	
Every day or nearly every day (or frequent drinking)	622 (18.9)	22.1 (19.8–24.6)	15.8 (13.6–18.2)	
In the past not now	184 (6.4)	6.0 (4.6–7.7)	6.9 (5.5–8.5)	
**Quantity ^b^ (Drink per occasion)**				<0.001
0 drinks	143 (6.9)	4.7 (3.4–6.4)	9.3 (7.4–11.6)	
1 drink	675 (29.1)	23.8 (21.0–26.8)	34.9 (31.7–38.3)	
2 drinks	735 (30.9)	31.1 (28.1–34.4)	30.7 (27.8–33.6)	
3 drinks	284 (12.3)	14.5 (12.3–17.0)	10.0 (8.3–12.1)	
4 drinks	157 (7.1)	8.7 (7.0–10.7)	5.4 (4.1–7.1)	
5 or more drinks	262 (13.7)	17.2 (14.7–20.0)	9.7 (7.5–12.3)	
**Heavy episodic drinking**	536 (19.5)	20.6 (18.2–23.2)	18.4 (16.0–21.0)	0.2
**Experiencing alcohol-related problems**	111 (3.6)	4.4 (3.3–5.7)	2.9 (2.2–4.0)	0.054
**Substance abuse disorder (SAD)**	34 (1.1)	0.7 (0.4–1.4)	1.5 (0.9–2.5)	0.07

Weighted percentages with 95% confidence intervals are reported. ^b^ Only current drinkers were included.

**Table 3:** Distribution of alcohol use measures by sociodemographic characteristics.

	Early Drinking Initiation W% (CI)	Frequent Drinking W% (CI)	HED W% (CI)	Experiencing Alcohol-Related Problems W% (CI)	SAD W% (CI)
**Age group**					
16–24	51.8 (44.7–58.9)	5.0 (2.6–9.6)	31.1 (24.7–38.3)	5.2 (3.0–8.9)	1.5 (0.4–5.0)
25–34	44.9 (39.2–50.9)	5.1 (3.2–8.1)	22.2 (17.6–27.8)	2.9 (1.6–5.2)	0.6 (0.2–1.9)
35–44	47.7 (42.5–53.0)	16.3 (13.2–20.0)	18.1 (14.7–22.0)	4.9 (3.2–7.3)	1.4 (0.5–3.6)
45–54	48.9 (43.6–54.3)	20.8 (17.1–25.1)	22.1 (18.4–26.3)	6.0 (4.3–8.4)	1.3 (0.6–2.8)
55–64	48.8 (44.3–53.3)	24.1 (20.3–28.3)	18.4 (14.9–22.5)	3.2 (1.9–5.3)	1.4 (0.7–2.7)
≥65	30.9 (27.5–34.6)	32.0 (28.5–35.7)	10.3 (8.2–13.0)	0.5 (0.2–1.3)	0.6 (0.3–1.5)
χ^2^ (*p*)	<0.0001	< 0.0001	<0.0001	<0.0001	0.7
**Ethnicity**					
European	51.0 (48.5–53.5)	26.3 (24.2–28.6)	18.3 (16.4–20.4)	3.8 (2.9–4.9)	1.0 (0.6–1.6)
Māori	55.7 (49.1–62.0)	10.0 (6.6–14.9)	32.5 (26.8–38.7)	4.9 (3.0–7.7)	3.0 (1.4–6.4)
Pacifica	28.7 (20.8–38.1)	4.9 (2.5–9.4)	33.9 (25.0–44.1)	7.3 (4.1–12.9)	1.4 (0.3–6.4)
Asian	20.7 (16.8–25.3)	3.7 (2.3–6.1)	8.2 (5.3–12.3)	0.5 (0.1–1.6)	NO
MELAA	24.5 (13.4–40.5)	5.8 (1.9–16.1)	9.6 (3.4–24.2)	1.9 (0.3–12.4)	NO
χ^2^ (*p*)	<0.0001	<0.0001	<0.0001	0.0003	0.02
**Having independent source of income**					
Yes	45.7 (43.3–48.1)	22.4 (20.5–24.4)	19.4 (17.5–21.3)	4.0 (3.2–4.9)	0.9 (0.6–1.4)
No	40.8 (35.8–45.9)	5.6 (3.9–8.1)	20.2 (16.1–24.9)	2.4 (1.4–4.0)	1.9 (0.9–4.0)
χ^2^ (*p*)	0.08	<0.0001	0.7	0.06	0.1
**Individual income**					
≥NZD 50K	51.3 (47.9–54.8)	24.0 (21.5–26.9)	19.8 (17.3–22.5)	4.0 (3.0–5.4)	0.4 (0.2–0.9)
<NZD 50K	40.8 (38.0–43.7)	15.6 (13.6–17.7)	19.4 (17.0–22.0)	3.5 (2.6–4.7)	1.7 (1.1–2.7)
χ^2^ (*p*)	<0.0001	<0.0001	0.8	0.5	0.0004
**Deprivation level**					
Low	48.6 (44.7–52.5)	25.9 (22.8–29.2)	17.2 (14.3–20.5)	3.4 (2.3–5.0)	0.5 (0.2–1.3)
Moderate	46.5 (43.2–49.7)	20.9 (18.3–23.7)	18.7 (16.3–21.2)	3.8 (2.7–5.2)	0.9 (0.4–1.7)
High	39.2 (35.0–43.6)	10.8 (8.7–13.4)	22.5 (19.0–26.4)	3.7 (2.6–5.2)	1.9 (1.1–3.3)
χ^2^ (*p*)	0.003	<0.0001	0.06	0.9	0.03
**Food security**					
Secure	45.2 (42.8–47.7)	21.3 (19.4–23.2)	18.2 (16.3–20.2)	3.2 (2.5–4.1)	0.7 (0.4–1.3)
Insecure	42.3 (37.5–47.3)	8.5 (6.2–11.4)	25.0 (20.9–29.6)	5.2 (3.5–7.5)	2.9 (1.8–4.7)
χ^2^ (*p*)	0.3	<0.00001	0.003	0.04	<0.0001

Weighted percentages with 95% confidence intervals are reported. NO: no observation.

**Table 4:** Age of regular drinking initiation by age cohort of participants.

Participant Age Group (Years)	Mean Age of Drinking Initiation (Years)
16–24	16.84
25–34	18.11
35–44	19.56
45–54	19.86
55–64	20.62
65+	23.43

On 1 December 1999, the legal age for purchasing alcohol was reduced from 20 years to 18 years.

**Table 5:** Association between alcohol use measures and adverse and positive health outcomes.

	Adverse Health Outcomes						Positive Health Outcomes			
	Chronic Health Condition		Poor Mental Health		Disability		Good General Health		Positive Mental Health	
	OR	AOR	OR	AOR	OR	AOR	OR	AOR	OR	AOR
Early drinking initiation (ref. = no)	0.85 (0.71–1.01)	0.93 (0.77–1.13)	1.05 (0.87–1.26)	0.91 (0.74–1.13)	0.98 (0.79–1.21)	1.00 (0.80–1.27)	0.97 (0.79–1.2)	0.89 (0.71–1.13)	0.96 (0.79–1.17)	0.95 (0.77–1.18)
Frequent drinking (ref. = no)	1.44 (1.18–1.75)	0.96 (0.76–1.22)	1.22 (0.99–1.50)	1.22 (0.97–1.55)	0.99 (0.75–1.31)	0.85 (0.63–1.15)	1.41 (1.08–1.84)	1.19 (0.89–1.59)	1.18 (0.94–1.49)	0.94 (0.73–1.21)
HED (ref. = no)	0.90 (0.73–1.11)	1.02 (0.80–1.31)	1.26 (1.00–1.58)	1.27 (0.99–1.64)	1.05 (0.81–1.37)	1.13 (0.84–1.52)	0.60 (0.47–0.77)	0.61 (0.47–0.81)	0.68 (0.55–0.85)	0.67 (0.53–0.84)
Experiencing alcohol-related problems	0.91 (0.60–1.38)	1.17 (0.75–1.81)	1.98 (1.31–3.00)	2.21 (1.42–3.46)	1.42 (0.88–2.27)	1.79 (1.06–3.00)	0.65 (0.41–1.02)	0.69 (0.43–1.01)	0.34 (0.22–0.52)	0.28 (0.18–0.42)
SAD	2.29 (1.04–5.02)	2.12 (0.89–5.08)	NO	NO	5.2 (2.28–11.67)	3.5 (1.42–8.62)	0.23 (0.10–0.52)	0.34 (0.15–0.80)	0.17 (0.07–0.38)	0.17 (0.8–0.39)

AOR: adjusted odds ratios, adjusted for gender, age groups, ethnicity, having an independent source of income, personal income, area deprivation level, and food security. NO: no observation.

**Table 6:** Association between early drinking initiation and alcohol-related problems.

	Frequent Drinking	HED	Experiencing Alcohol-Related Problems	SAD
Early drinking initiation				
OR	1.36 (1.11–1.66)	3.77 (2.99–4.76)	3.45 (2.17–5.49)	0.74 (0.30–1.81)
AOR	1.14 (0.92–1.42)	3.24 (2.50–4.19)	2.49 (1.51–4.12)	0.63 (0.26–1.50)

OR: odds ratio. AOR: adjusted odds ratio, adjusted for gender, age groups, ethnicity, having an independent source of income, personal income, area deprivation level, and food security. HED: heavy episodic drinking; SAD: substance abuse disorder.

## Data Availability

The research data presented in this article are not readily available due to the confidentiality and sensitivity of the data and Māori data sovereignty.

## Supplementary Materials

The following supporting information can be downloaded at: https://www.mdpi.com/article/10.3390/healthcare12161592/s1, Table S1: Definition of socioeconomic status, alcohol measures, and health outcome variables (positive and negative physical and mental health, and disability), He Koiora Matapopore.
