# Supplementary material for: Population-Level Impacts of Alcohol Use on Mental and Physical Health Outcomes
Source: Healthcare (Basel). 2024 Aug 9;12(16):1592. doi: 10.3390/healthcare12161592 (PMC11353263; doi:10.3390/healthcare12161592)
Supplement: Supplementary file 1 [file healthcare-12-01592-s001.zip › healthcare-3072171-supplementary.pdf]

Table S1: Definition of socioeconomic status, alcohol measures, and health outcome variables (positive and negative physical and mental health, disability), He Koiora Matapopore

Variable	Definition
**Demographic characteristics**	
Having an independent source of income	Respondents were asked whether they had access to independent income from wages, investments, or retirement income.
Food security status	Respondents were asked whether they have ever been worried about not having enough money to buy food. We scored responses of "never" as 0 and all other responses (Occasionally/Sometimes/Often/All the time) as 1.
Area-level deprivation (Indices of Multiple Deprivation (IMD))	Taken from NZ index of multiple deprivation (IMD) [20] which used a combination of routinely collected data from government departments and census data in seven domains (i.e. employment, income, crime, housing, health, education, and access to services) to develop a measure of deprivation at the neighborhood level. Participants were classified in three groups: living in low, moderate and high deprived area.
**Alcohol-related measures**	
Early drinking initiation	Respondents were asked "How old were you when you started drinking regularly?" Early drinking initiation was defined as starting drinking before the age of 18 years. Age 18 is the legal age at which people can purchase alcohol in New Zealand.
Frequency of drinking	Respondents were asked "How often do you drink alcohol?" Response options were: Never, Less than once a month, 1-3 times in a month, Once to twice a week, Every day or nearly every day, In the past, not now. Frequent drinking was defined as drinking every day or nearly every day.
Drink per occasion	Only those who indicated at the time of interview were "asked: "On the days that you drank in the past FOUR weeks, about how many alcoholic drinks did you usually have a day? Response options were 0,1,2,3,4,5 or more.
Heavy Episode Drinking (HED)	HED (derived variable) was defined (following the New Zealand Health Promotion Agency guideline [13]) as four or more drinks (for female respondents) or five or more (for male respondents) on the days that respondent drank in the past four weeks.
Experiencing alcohol-related problems	Respondents were asked "In the past 12 months, have you experienced any of the following problems, related to your drinking?" Money problems, health problems, conflict with family and friends, problems with authorities (police, bar owner, etc.), other problems. A binary variable was created for experiencing any alcohol-related problem versus none.
Substance abuse disorder (SAD)	Respondents were asked "Have you ever been told by your doctor that you have Substance abuse disorder, including alcohol abuse?".
**Health outcomes**	
**Physical health**	
General health	Respondents were asked whether, in general, they describe their overall health as excellent, good, fair, poor, or very poor? Responses were categorized as poor (Fair, poor, or very poor) or good (good or excellent).
Chronic health condition	Respondents were asked whether they have ever been told by their doctor that they have any of the following long-term health conditions? a)Heart disease including heart attack, angina, or heart failure, b) Cancer, c) Stroke, d) Diabetes (not

Variable	Definition
	including gestational diabetes), e) Asthma, f) Arthritis, including gout, lupus, and psoriatic arthritis, g) others including Endocrine disease, Musculoskeletal disease, ENT (Ear, Nose & Throat) disease, Ophthalmology Eye disease, Neurological disease, Gastrointestinal disease, Genitourinary Disorder, Respiratory disease, Metabolic disease. A binary variable was also created for reporting any (at least one) physical health condition versus none.
**Disability**	A binary variable was created for reporting at least one type of disability versus none.<br>Respondents were asked whether they had a long-term health problem or a condition (lasted 6 months or longer) that caused them difficulty with, or stopped them from:
Physical disability	seeing, hearing, walking, lifting or bending, using their hands to hold, grasp, or use objects
Psychological disability	communicating, mixing with others or socializing
Cognitive disability	learning, concentrating, or memorizing
**Mental health**	
Mental health condition	Respondents were asked whether they have been ever told by their doctor that they have any of the following long-term mental health conditions? a) Depression, b) Anxiety, c)Other (including PTSD, OCD (Obsessive-Compulsive Disorder), Insomnia, Schizophrenia etc.).
Taking antidepressant medication	Respondents were asked whether in the past four weeks, they have taken medication to help them not feel sad or depressed? (yes/no)
Taking sleep medication	Respondents were asked whether in the past four weeks, they have taken medication to help them calm down or sleep? (yes/no)
Poor mental health	Respondents were classified as having low mental health if they had received a diagnosis of aforementioned mental health conditions or reported taking sleep or antidepressant medication in the past four weeks.
Positive mental health	Keyes' Mental Health Continuum Short Form (MHC-SF) was used to measure positive mental health. The MHC-SF groups 13 dimensions of mental health under three categories: Positive Emotions (including positive affect and avowed quality of life), Positive Psychological Functioning (including self-acceptance, personal growth, purpose in life, environmental mastery, autonomy and positive relations with others), and Positive Social Functioning (including social acceptance, social actualization, social contribution, social coherence and social integration) [11]. Participants rated the frequency with which they experienced each of these items in the past month on a 6-point Likert scale, where never=1, every day=6. Positive mental health was defined as having a score of 5 or 6 on one of measure of emotional well-being, and a score of 5 or 6 on six of the eleven items of psychological and social wellbeing.
